# Effects of indacaterol/glycopyrronium (QVA149) on lung hyperinflation and physical activity in patients with moderate to severe COPD: a randomised, placebo-controlled, crossover study (The MOVE Study)

**DOI:** 10.1186/s12890-016-0256-7

**Published:** 2016-06-14

**Authors:** Henrik Watz, Claudia Mailänder, Monika Baier, Anne Kirsten

**Affiliations:** Pulmonary Research Institute at Lung Clinic Grosshansdorf, Airway Research Center North, Member of the German Center for Lung Research, Woehrendamm 80, D-22927 Grosshansdorf, Germany; Novartis Pharma GmbH, Nuremberg, Germany

**Keywords:** COPD, Physical activity, Bronchodilator, Hyperinflation

## Abstract

**Background:**

Physical activity limitation is common in chronic obstructive pulmonary disease (COPD), and is associated with worse health status, and increased hospitalisation and mortality. Long-acting bronchodilators, either alone or in combination, have been shown to improve exercise intolerance. However, none of these studies were designed with physical activity as primary outcome.

This study assessed the effect of indacaterol/glycopyrronium fixed dose combination (IND/GLY) 110/50 μg once daily (OD) versus placebo on lung hyperinflation (inspiratory capacity [IC]) and physical activity in patients with moderate-to-severe COPD.

**Methods:**

In this multicentre, randomised, double-blind, placebo-controlled crossover study, patients received IND/GLY or placebo OD in two 21-day treatment periods (14-day washout between periods). Eligible patients were ≥40 years of age, current or ex-smokers (smoking history ≥10 pack-years), with post-salbutamol forced expiratory volume in 1 s (FEV_1_) 40–80 % predicted, and FEV_1_:forced vital capacity <0.70.

The co-primary endpoints were peak IC after 21 days and average daily activity-related energy expenditure. Key secondary endpoints were average number of steps per day and the duration of at least moderate activity per day. Peak IC and FEV_1_ on Day 1, and trough IC and FEV_1_ after 21 days were other secondary endpoints.

**Results:**

A total of 194 patients were randomised (65.5 % male, mean age 62.8 years, mean FEV_1_ 61.6 % predicted), with 183 (94.3 %) completing the study.

Compared with placebo, IND/GLY significantly increased peak IC after 21 days (difference 202 mL, *p* < 0.0001), activity-related energy expenditure (difference 36.7 kcal/day, *p* = 0.040), and the average number of steps per day (difference 358, *p* = 0.029), with a trend towards an improvement in the duration of at least moderate activity (difference 4.4 min, *p* = 0.264). IND/GLY was associated with statistically significant improvements versus placebo in peak IC and FEV_1_ on Day 1, and trough IC and FEV_1_ after 21 days. The incidence of treatment-emergent adverse events was 22.8 % with IND/GLY and 22.9 % with placebo.

**Conclusions:**

In this study, compared with placebo, IND/GLY reduced hyperinflation, and, despite no patient education or lifestyle advice, improved daily physical activity levels. This suggests that IND/GLY has the potential to impact two of the main clinical concerns in the care of patients with COPD.

**Trial registration:**

ClinicalTrials.gov number: NCT01996319.

**Electronic supplementary material:**

The online version of this article (doi:10.1186/s12890-016-0256-7) contains supplementary material, which is available to authorized users.

## Background

Limitation of physical activity is common in chronic obstructive pulmonary disease (COPD) [1]. Recent data suggest that patients with COPD are three times more likely than individuals without COPD to report activity limitation, and three times more likely to have difficulty walking or climbing stairs [2]. The importance of maintaining or improving physical activity levels in patients with COPD is increasingly recognised, as low or decreasing levels of physical activity are associated with a worsening in health status, [3] and increasing rates of hospitalisation [4] and mortality [4, 5]. Furthermore, sustained physical inactivity is also associated with a decline in muscle mass, [3] which will, in turn, impact the ability of patients to exercise.

One of the main reasons for activity limitation could be lung hyperinflation, which not only affects patients with advanced COPD, but can also occur early in the course of the disease [6]. A number of long-acting bronchodilators have been shown to reduce lung hyperinflation and thereby improve exercise intolerance, including the long-acting β_2_-agonist (LABA) indacaterol, [7, 8] the long-acting muscarinic antagonist (LAMA) glycopyrronium, [9] and the fixed-dose combination of indacaterol and glycopyrronium (IND/GLY; QVA149) [10]. The improvements in exercise intolerance due to lung deflation by bronchodilator therapy have been frequently studied using standardised protocols [7–11]. However, improved exercise tolerance only permits increased physical activity – it does not mean that patients will change their behaviour and become more physically active [12]. Despite this, given the close relation between physical inactivity and unfavourable patient outcomes, it is the goal of such interventions to also improve physical activity in patients with COPD [1, 13]. Unfortunately, few randomised, placebo-controlled studies have evaluated the effects of such therapies on objectively measured physical activity in patients with COPD. Those that have been conducted provided mixed results with regard to an improvement of physical activity, [8, 14–16] although none of these studies were designed with physical activity as the primary outcome of the intervention.

The purpose of this study was to assess the effect of IND/GLY 110/50 μg once daily (OD) versus placebo on lung hyperinflation as assessed by the measurement of inspiratory capacity (IC) and objectively measured physical activity in patients with moderate-to-severe COPD.

## Methods

### Trial design

**Fig. 1:**
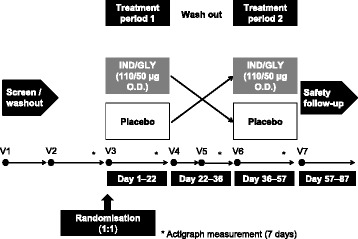
Study design

This was a multicentre, randomised, double-blind, placebo-controlled crossover study, conducted at 30 secondary care (pulmonology) practices in Germany. It consisted of two 21-day treatment periods, with a 14-day washout between periods (Fig. 1).

At an initial screening visit (Visit 1), patients provided informed consent, inclusion/exclusion criteria were checked and their current COPD medication was reviewed and (if necessary) adjusted. The period between Visit 1 and Visit 2 was an individual run-in period, the duration of which depended on COPD medication washout. At Visit 2 (the first baseline visit), inclusion/exclusion criteria were reassessed, including spirometry before and after inhalation of salbutamol 400 μg. Treatment Period 1 ran for 21 days, commencing with the randomisation visit (Visit 3), when eligible patients were randomised to one of two treatment sequences, and ending on Visit 4. This was followed by a 14-day washout period, which included the second baseline visit (Visit 5) on Day 29. Treatment period 2 then ran for 21 days from Visit 6 to Visit 7.

Physical activity was measured with an activity monitor that has been validated for use in COPD (Sensewear® armband; BodyMedia Inc., Pittsburgh, PA, USA) [17, 18]. Patients were to wear the armband for the week (e.g., Monday to Monday) prior to the start of each treatment period (to collect baseline data), and for the last week of each treatment period. The armband was to be worn continuously day and night, except for time spent in personal hygiene. We assessed activity-related energy expenditure (i.e., total daily energy expenditure minus resting energy expenditure), minutes of at least moderate activity (>3 metabolic equivalents), steps per day, and physical activity level (total daily energy expenditure divided by resting energy expenditure) as previously described [19, 20]. We defined a valid day to have at least 21.5 h of monitoring (i.e., 90 % of the potential wearing time). The first day of each measurement period was not used for analysis as the measurement either started after the visit at the study site (baseline) or a reminder phone call from the study nurse (treatment period), resulting in highly variable wearing times on that day [20]. The last day of each measurement period, when the patients had to come to the study site for the respective visit in the morning, was also not used, resulting in 6 days (each of 1440 min) of measurement [14, 15, 20]. On Visits 3, 4, 6 and 7, IC was assessed from slow vital capacity (SVC) spirometry manoeuvres at 30 min predose, and 15 and 45 min and 1.5, 2.5 and 3.5 h post-dose; forced expiratory volume in 1 s (FEV_1_) was assessed from forced vital capacity (FVC) manoeuvres at 15 min predose, and 30 min and 1, 2, 3 and 4 h post-dose. Lung function was assessed according to American Thoracic Society/European Respiratory Society standards, [21] using established reference values for FEV_1_ [22] and suggested reference values for IC [23].

Patients were to be withdrawn from the study if they experienced a moderate or severe COPD exacerbation. Adverse events (AEs) and serious AEs (SAEs) were collected throughout the study, with vital signs collected at each visit.

There was one substantial protocol amendment, to add an exclusion criterion (glomerular filtration rate <50 mL/min/1.73 m^2^) and a laboratory parameter (creatinine clearance), and to correct some inconsistencies. A nonsubstantial amendment reflected a change from electronic to paper case report forms. The study was conducted in accordance with the Declaration of Helsinki.

### Participants

Eligible patients were male or female, at least 40 years of age, current or ex-smokers with smoking history of at least 10 pack-years, and had signed an informed consent form prior to initiation of any study-related procedure. All patients were to have stable COPD, with post-bronchodilator (400 μg salbutamol) FEV_1_ between 40 and 80 % of predicted normal and FEV_1_/FVC <0.70 at Visit 2.

The main exclusion criteria were concomitant pulmonary disease, history of asthma, onset of respiratory symptoms prior to age 40 years, blood eosinophil count >600/mm^3^ during run-in, or a clinically significant abnormality that could interfere with the assessment of efficacy or safety of the study. Patients were also excluded if they experienced a COPD exacerbation in the 6 weeks prior to screening or during the run-in period, or a respiratory tract infection within 4 weeks prior to screening or during the run-in period, or had a contraindication for, or hypersensitivity to, anticholinergics, β_2_-agonists, sympathomimetic amines, or lactose or any of the other excipients of the study medication. Participation in, or planning participation in, a pulmonary rehabilitation programme was also a reason for exclusion.

Consistent with similar studies, [14, 15] the following COPD medication was prohibited from the indicated time prior to Visit 2 and for the duration of the study: LAMAs (7 days); LABAs (48 h; 7 days for indacaterol); xanthines and oral phosphodiesterase IV inhibitors (7 days). Inhaled corticosteroids (ICSs) were permitted, at a stable dose throughout the study (patients on a LABA/ICS combination were to be switched to the nearest equivalent dose of ICS monotherapy at least 48 h prior to Visit 2).

### Interventions

Study treatment was IND/GLY, at a dose of 110/50 μg OD, and matching placebo, with patients, investigators and staff, persons performing the assessments, sponsor staff and data analysts blinded to treatment. Salbutamol was permitted as rescue medication, but not within 6 h of the start of any study visit.

Patients were randomised to one of two treatment sequences in a ratio of 1:1 using a randomisation number from a list generated by the study sponsor. This list was produced using a validated automated system.

### Outcomes and statistical methods

The two co-primary objectives were: To demonstrate the superiority of IND/GLY to placebo in terms of peak IC after 21 days of treatment; and to evaluate the superiority of IND/GLY to placebo in terms of average daily activity-related energy expenditure. Both co-primary objectives were analysed using an analysis of covariance (ANCOVA) model, with the factors centre, baseline measure, period, patient within centre, and treatment. Multiplicity was controlled using the Bonferroni-Holm procedure, in which the two hypotheses were tested at the 2.5 % level in a first step. If at least one of the hypotheses could be rejected at this level, the remaining hypothesis was then tested at the 5 % level. Data for the two co-primary objectives are presented as adjusted (least squares) mean treatment differences, two-sided 95 % confidence intervals and associated p values. *Post-hoc* analyses were performed to identify potential subgroups that were more likely to respond to IND/GLY treatment with regard to changes of activity-related energy expenditure.

Key secondary objectives were to evaluate the effects of IND/GLY vs placebo in terms of average number of steps per day and the duration of at least moderate activity per day. Other secondary objectives were comparisons of IND/GLY with placebo in terms of peak IC and FEV_1_ on Day 1, and trough IC and FEV_1_ after 21 days, with average physical activity level analysed as an exploratory endpoint. The secondary and exploratory efficacy objectives were analysed using a similar ANCOVA model to that used for the co-primary objectives, with the exception of steps per day. This was analysed using Friedman’s test for paired data, with results presented as mean and standard deviation (SD) and associated p value. Use of rescue medication was also captured during the study, and the number of days on which rescue medication was used was calculated (expressed as per 100 study days).

The full analysis set consisted of all randomised patients who were exposed to at least one dose of study medication in one treatment period, and was used for all efficacy analyses. The safety set consisted of all randomised patients who were exposed to at least one dose of study medication in one treatment period and had at least one safety assessment; this was used for all safety analyses.

### Sample size

In a previous study, the mean difference between indacaterol and placebo for change from baseline in average daily energy consumption was 132.6 Kcal/day, with a within-patient standard deviation (SD) of 389.3 Kcal/day [15]. It was estimated that to observe this difference, 92 patients were needed in a crossover design. However, in order to observe significant differences in the other co-primary endpoint, IC, and in the other two activity parameters (steps per day and duration of at least moderate activity), a sample size was selected based on the parameter with the smallest effect size (observed mean indacaterol–placebo difference of 711.5 steps per day with a within-patient SD of 2679.7 steps per day [15]). For this parameter, 150 patients would give 90 % power with a two-sided 5 % significance level. It was estimated that this would require approximately 190 patients to be randomised.

## Results

### Participants

**Fig. 2:**
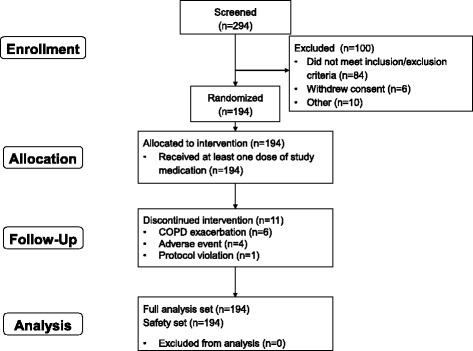
Patient flow through the study

Of 294 patients screened, 194 were randomised, all of whom were exposed to at least one dose of study treatment; all randomised patients were included in both the safety and full analysis sets (Fig. 2). A total of 191 patients completed the first treatment period, and 183 (94.3 %) completed the study. The baseline demographics and disease characteristics of the randomised patients are shown in Table 1. Mean overall compliance was 99.5 % to IND/GLY and 99.6 % to placebo. The mean wearing time for the Sensewear device during IND/GLY treatment was 1412.8 and 1413.0 min per day (both 98.1 % of the maximum) at baseline and the end of the treatment period, respectively, and 1411.6 and 1411.5 min per day (both 98.0 %) during placebo treatment. Corresponding mean number of days worn were 5.8 and 5.8 for IND/GLY, and 5.9 and 5.7 for placebo.

**Table 1:** Baseline demographics and disease characteristics (full analysis set)

Parameter	Total (*N* = 194)
Sex, male, n (%)	127 (65.5)
Race, n (%)	
Caucasian	187 (96.4)
Other	7 (3.6)
Age, years, mean (SD)	62.8 (7.9)
BMI, kg/m^2^, mean (SD)	26.7 (4.7)
Smoking status, n (%)	
Ex-smoker	84 (43.3)
Current smoker	110 (56.7)
Pack-years, mean (SD)	47.5 (21.5)
Severity of COPD (GOLD 2010), n (%)	
Stage I	1 (0.5)
Stage II	160 (82.5)
Stage III	32 (16.5)
Not assessable	1 (0.5)
Post-bronchodilator FEV_1_, % predicted, mean (SD)	61.6 (10.7)
Post-bronchodilator FEV_1_/FVC, %, mean (SD)	51.2 (9.1)
COPD Assessment Test, mean (SD)	15.9 (6.0)

*SD* standard deviation, *BMI* body mass index, *COPD* chronic obstructive pulmonary disease, *GOLD* Global Initiative for Chronic Obstructive Lung Disease, *FEV*_*1*_, forced expiratory volume in 1 s, *FVC* forced vital capacity

### Outcomes

#### Co-primary endpoints

**Fig. 3:**
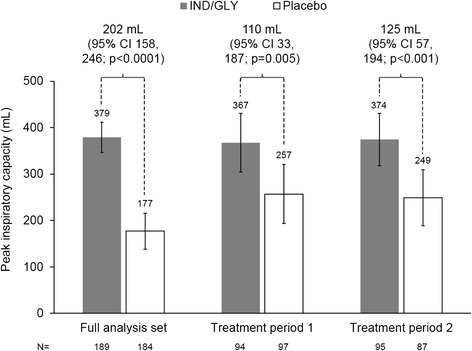
LS mean change from baseline in peak inspiratory capacity after 21 days, overall and for each treatment period (full analysis set)

**Fig. 4:**
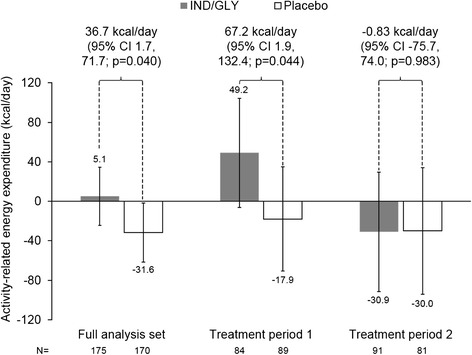
LS mean change from baseline in activity-related energy expenditure, overall and for each treatment period (full analysis set)

Compared with placebo, IND/GLY was associated with a statistically significant increase in both peak IC after 21 days and activity-related energy expenditure (Figs. 3 and 4).

Significant IND/GLY–placebo differences were observed for peak IC in both treatment periods (Fig. 3). For energy expenditure, although there was a significant difference between IND/GLY and placebo in Treatment Period 1, there was no difference between treatments in period 2, with a decrease from baseline in energy expenditure in both groups (Fig. 4). As shown by the raw mean data in Additional file 1: Figure S1, the baseline energy expenditure value for the placebo group in Treatment Period 2 (the group who received IND/GLY in Treatment Period 1) was higher than either of the on-treatment IND/GLY mean values. Raw mean data for peak IC are shown in Additional file 1: Figure S2.

*Post-hoc* analyses of changes in activity-related energy expenditure according to baseline COPD Assessment Test (CAT) score, age, gender, baseline activity levels, and smoking status did not identify any subgroups that were more likely to respond to IND/GLY treatment, with the *p* values for the interaction within all groups being >0.05. However, when the patient population of this study was subgrouped according to the median baseline post-bronchodilator IC, activity-related energy expenditure was significantly increased with IND/GLY vs placebo in the subgroup with baseline IC <72.9 % predicted (*p* = 0.004), but not IC ≥72.9 % (Additional file 1: Figure S3). Similarly, activity-related energy expenditure was significantly increased with IND/GLY vs placebo in the more severe FEV_1_ subgroup (FEV_1_ < 62.2 % predicted; *p* = 0.005), but not in the less severe group (Additional file 1: Figure S3).

#### Key secondary endpoints

The key secondary endpoints, duration of at least moderate activity and average number of steps per day, are shown in Table *2*. There was a significant improvement in the average number of steps per day with IND/GLY compared with placebo. For the duration of at least moderate activity, there was a decrease from baseline with both treatments, although the decrease was smaller with IND/GLY than placebo, resulting in a trend towards an improvement with IND/GLY vs placebo, although this difference didn’t reach statistical significance.

**Table 2:** Actigraphy endpoints (full analysis set)

Endpoint	Treatment	Baseline value, mean	Change from baseline	
			LS mean (95 % CI)^a^or mean (SD)^b^	LS mean difference (95 % CI)^a^or mean (SD)^b^
Duration of at least moderate activity per day, minutes	IND/GLY	125	-6.9 (-13.4, -0.4)	4.4 (-3.3, 12.1; *p* = 0.264)
	Placebo	130	-11.3 (-17.9, -4.6)	
Average number of steps per day	IND/GLY	5602	31 (1662.4)	358 (2458.0; *p* = 0.029)
	Placebo	5849	-321 (1647.6)	
Average physical activity	IND/GLY	1.49	0.01 (0, 0.03)	0.02 (0, 0.04); *p* = 0.019
	Placebo	1.50	-0.01 (-0.03, 0.01)	

^a^Duration of at least moderate activity and average physical activity

^b^Average number of steps per day

#### Secondary and exploratory endpoints

The secondary endpoints are shown in Table 3, with the average physical activity exploratory endpoint in Table *2*. IND/GLY was associated with statistically significant improvements compared with placebo for all of these endpoints. Use of rescue medication was higher during treatment with placebo (1981 days; 47.8 per 100 study days) than with IND/GLY (1188 days; 27.7 per 100 study days).

**Table 3:** Spirometry endpoints (full analysis set)

Endpoint	Treatment	Baseline value, mean	Change from baseline	
			LS mean (95 % CI)	LS mean difference (95 % CI)
Peak IC on Day 1, L	IND/GLY	2.36	0.46 (0.42, 0.49)	0.26 (0.22, 0.30); *p* < 0.0001
	Placebo	2.39	0.20 (0.17, 0.24)	
Trough IC after 21 days, L	IND/GLY	2.36	0.11 (0.07,0.16)	0.20 (0.15, 0.25); *p* < 0.0001
	Placebo	2.40	-0.09 (-0.13, -0.04)	
Peak FEV_1_ on Day 1, L	IND/GLY	1.57	0.34 (0.31, 0.37)	0.22 (0.19, 0.25); *p* < 0.0001
	Placebo	1.59	0.12 (0.09, 0.15)	
Peak FEV_1_ after 21 days, L	IND/GLY	1.57	0.42 (0.39, 0.45)	0.37 (0.33, 0.40; *p* < 0.0001)
	Placebo	1.59	0.05 (0.02, 0.09)	
Trough FEV_1_ after 21 days, L	IND/GLY	1.57	0.20 (0.17, 0.23)	0.28 (0.24, 0.31); *p* < 0.0001
	Placebo	1.60	-0.08 (-0.11, -0.05)	

### Safety

Treatment-emergent AEs (TEAEs) are shown in Table 4. The only suspected related TEAE to occur in more than one patient in either group was cough (in 5 [2.6 %] patients receiving IND/GLY, and 1 [0.5 %] patient receiving placebo). Six patients discontinued study drug due to TEAEs, four receiving IND/GLY and two receiving placebo. Of these, four also discontinued the study due to AEs, three after receiving IND/GLY and one after receiving placebo. The other two discontinued the study due to an exacerbation (as required by the protocol), one after receiving IND/GLY and one after placebo. One death occurred: a suspected myocardial infarction, 4 days after the last dose of IND/GLY. This was not considered by the investigator to be related to study treatment, with the investigator reporting no signs of ischaemic disease at the last visit (4 days prior to death).

**Table 4:** Treatment-emergent AEs, overall and by preferred term (≥2.5 % with either treatment) (safety set)

	IND/GLY (*N* = 193)		Placebo (*N* = 188)	
	Events, n	Patients, n (%)	Events, n	Patients, n (%)
All TEAEs	73	44 (22.8)	48	43 (22.9)
Cough	8	8 (4.1)	3	3 (1.6)
Nasopharyngitis	7	7 (3.6)	8	8 (4.3)
Headache	7	6 (3.1)	3	3 (1.6)
Suspected related TEAEs	12	11 (5.7)	4	4 (2.1)
Cough	5	5 (2.6)	1	1 (0.5)
Serious TEAEs	4	4 (2.1)	2	2 (1.1)
Deaths	1	1 (0.5)	0	0
Suspected related serious TEAE	0	0	0	0

*TEAE* treatment-emergent adverse event

Overall, there was a low incidence of newly occurring or worsening of notable abnormal vital signs values with no clinically meaningful differences between the treatments. All affected abnormal vital signs referred to the blood pressure and most notable abnormal vital signs values were only borderline increased or decreased.

## Discussion

COPD is characterised by a substantial decrease in physical activity across all severity stages, with the main cause being breathlessness due to hyperinflation. It is therefore of clinical interest that the two co-primary endpoints in this study were met, with statistical separations between IND/GLY and placebo in terms of peak IC (i.e., hyperinflation) and activity-related energy expenditure. These significant improvements with IND/GLY vs placebo were accompanied by a significant improvement in the average number of steps per day, and a trend towards an improvement in the duration of at least moderate activity (although this difference didn’t reach statistical significance).

The difference in peak IC (202 mL) exceeded the value of 100 mL that has been suggested to be potentially clinically relevant [24]. The two treatment periods gave consistent results for this parameter, indicating that the washout period was sufficient for the spirometry endpoints. There was, however, a substantial period effect in activity-related energy expenditure. In the first period, average energy expenditure increased from baseline in patients receiving IND/GLY. In Period 2, there was a decrease from baseline with both treatments. Notably, however, the baseline value in Period 2 of patients receiving placebo was higher than the other three baseline evaluations. As these were patients who received IND/GLY in the first treatment period this suggests that the 3-week treatment period may have been insufficient to fully evaluate the effect of IND/GLY on physical activity. It would be intriguing to evaluate the longer-term effect of IND/GLY on this parameter.

It should be recognised that the only intervention that patients received in the current study was pharmacological (either IND/GLY or placebo). Reducing hyperinflation with a long-acting bronchodilator has consistently been shown to increase exercise capacity [7–11, 14, 25, 26]. However, just increasing the ability of patients to exercise doesn’t mean that they will make use of this additional capacity, [12] as shown in an indacaterol study in which despite an increase in exercise endurance of nearly 2 min (assessed using cycle ergometry) with indacaterol 300 μg OD compared with placebo, average energy expenditure and physical activity duration was slightly lower with indacaterol than placebo [8]. In contrast, in studies that assessed physical activity using the same standardised methodology as in the current study, significant differences were observed versus placebo in favour of indacaterol 150 μg OD, [15] and aclidinium 400 μg BID [14]. Even so, the improvements in physical activity observed in all of these studies are less than observed with behavioural interventions and patient education, the importance of which is increasingly recognised [1]. Indeed, patient education forms an important part of recommended pulmonary rehabilitation programmes [27–30]. Furthermore, the combination of a long-acting bronchodilator (tiotropium) and pulmonary rehabilitation has been shown to produce sustained improvements in breathlessness and health status [31]. However, such behavioural interventions can be very challenging to incorporate into clinical trials – as demonstrated by a tiotropium study in which, despite complex individualised patient education and goals setting there were no statistically significant differences between tiotropium and placebo for any of the activity endpoints [16, 32]. In this context, it is notable that IND/GLY was associated with significant improvements in a range of physical activity parameters in the current trial.

The study was powered on number of steps, and it is important, therefore, that there was a significant improvement with IND/GLY over placebo for this parameter. A previous study has suggested that every additional 1000 steps per day at low average intensity is associated with a 20 % decrease in the risk of COPD hospitalisation [33]. Although the 358 step difference between treatments in the current study is one-third of this value, this demonstrates the potential for a longer treatment intervention, perhaps in combination with a behavioural intervention, to have a significant impact on hospitalisation. This relatively short duration is one of the main weaknesses of the study. The crossover design is both a strength and a weakness; as patients act as their own controls, the variability of data is anticipated to be lower, permitting a smaller study. However, interpreting data from the second treatment period is especially challenging, since the physical activity results depend on a change in lifestyle. In addition, the population was not recruited based on their baseline hyperinflation or level of physical activity – and there was a high degree of variability in baseline physical activity levels, with the duration of at least moderate activity varying from 3 to 498 min. Furthermore, the *post-hoc* analyses suggest that the effects of IND/GLY on physical activity in this study are mainly driven by patients with more severe COPD. This hypothesis, however, would be clearly subject to further prospective trials and is not in line with our previous study using indacaterol in patients with a very similar degree of lung function impairment [15].

The finding that the duration of at least moderate activity decreased with both treatments is unexpected, especially given that the average steps per day and physical activity increased as a result of treatment with IND/GLY. However, this group of patients appeared to have fairly high durations of physical activity on entry – mean baseline values of 125 and 130 mins per day, which is about 30 % higher than in previous studies with similar COPD populations [14, 15]. This would suggest that the patients in the current study had limited opportunity within their day-to-day lifestyle to increase the duration of physical activity.

As would be anticipated from previous studies, all of the secondary spirometry parameters were significantly increased with IND/GLY compared with placebo. The difference between IND/GLY and placebo for trough FEV_1_ after 21 days (280 mL) was consistent with that observed in an exercise endurance study (200 mL), [10] and for the primary endpoint (at Week 26) of the pivotal registration study by Bateman et al (a difference of 200 mL) [34]. The difference in trough IC after 21 days in the current study (200 mL) was also similar to the prior exercise endurance study (190 mL) [10]. Consistent with previous studies, IND/GLY also demonstrated a good overall safety profile, with no unexpected findings in either the AEs or the serious AEs.

## Conclusion

In conclusion, in this study IND/GLY was associated with significant reductions compared with placebo in hyperinflation, and, despite no patient education or lifestyle advice, significant improvements in daily physical activity levels. This suggests that IND/GLY has the potential to impact two of the main clinical concerns in the care of patients with COPD. However, longer trials combined with either behavioural education or pulmonary rehabilitation are needed to demonstrate clinically more relevant effects on physical activity.

## Abbreviations

AE, adverse event; ANCOVA, analysis of covariance; BMI, body mass index; CAT, COPD Assessment Test; CI, confidence interval; COPD, chronic obstructive pulmonary disease; FEV_1_, forced expiratory volume in 1 s; FVC, forced vital capacity; GOLD, Global Initiative for Chronic Obstructive Lung Disease; IC, inspiratory capacity; ICS, inhaled corticosteroid; IND/GLY, fixed-dose combination of indacaterol and glycopyrronium (QVA149); LABA, long-acting β_2_-agonist; LAMA, long-acting muscarinic antagonist; OD, once daily; SAE, serious adverse event; SD, standard deviation; SVC, slow vital capacity; TEAE, treatment-emergent adverse event.

## Additional file

Additional file 1: Figure S1. Raw mean peak inspiratory capacity after 21 days, overall and for each treatment period (full analysis set). Figure presenting peak inspiratory capacity data, for the full analysis set and for treatment periods 1 and 2. **Figure S2.** Raw mean activity-related energy expenditure, overall and for each treatment period (full analysis set). Figure presenting activity-related energy expenditure, for the full analysis set and for treatment periods 1 and 2. **Figure S3.** LS mean change from baseline in activity-related energy expenditure, by median baseline FEV_1_ and IC (full analysis set). Figure presenting the change from baseline in activity related energy expenditure for four subgroups: Patients with baseline FEV_1_ < 62.2 % and ≥62.2 % (the median baseline value was 62.2 %); and patients with baseline IC <72.9 % and ≥72.9 % (the median baseline value was 72.9 %). (PDF 259 kb)

## References

[1] Watz H, Pitta F, Rochester CL, Garcia-Aymerich J, ZuWallack R, Troosters T, Vaes AW, Puhan MA, Jehn M, Polkey MI, Vogiatzis I, Clini EM, Toth M, Gimeno-Santos E, Waschki B, Esteban C, Hayot M, Casaburi R, Porszasz J, McAuley E, Singh SJ, Langer D, Wouters EFM, Magnussen H, Spruit MA (2014). An official European Respiratory Society statement on physical activity in COPD. Eur Respir J.

[2] Wheaton AG, Cunningham TJ, Ford ES, Croft JB (2015). Employment and activity limitations among adults with chronic obstructive pulmonary disease--United States, 2013. MMWR Morb Mortal Wkly Rep.

[3] Waschki B, Kirsten AM, Holz O, Mueller K-C, Schaper M, Sack A-L, Meyer T, Rabe KF, Magnussen H, Watz H (2015). Disease progression and changes in physical activity in patients with chronic obstructive pulmonary disease. Am J Respir Crit Care Med.

[4] Garcia-Aymerich J, Lange P, Benet M, Schnohr P, Antó JM (2006). Regular physical activity reduces hospital admission and mortality in chronic obstructive pulmonary disease: a population based cohort study. Thorax.

[5] Waschki B, Kirsten A, Holz O, Müller K-C, Meyer T, Watz H, Magnussen H (2011). Physical activity is the strongest predictor of all-cause mortality in patients with COPD: a prospective cohort study. Chest.

[6] Ofir D, Laveneziana P, Webb KA, Lam Y-M, O’Donnell DE (2008). Mechanisms of dyspnea during cycle exercise in symptomatic patients with GOLD stage I chronic obstructive pulmonary disease. Am J Respir Crit Care Med.

[7] Beeh K-M, Wagner F, Khindri S, Drollmann AF (2011). Effect of indacaterol on dynamic lung hyperinflation and breathlessness in hyperinflated patients with COPD. COPD.

[8] O’Donnell DE, Casaburi R, Vincken W, Puente-Maestu L, Swales J, Lawrence D, Kramer B, for the INABLE 1 study group (2011). Effect of indacaterol on exercise endurance and lung hyperinflation in COPD. Respir Med.

[9] Beeh KM, Singh D, Di Scala L, Drollmann A (2012). Once-daily NVA237 improves exercise tolerance from the first dose in patients with COPD: the GLOW3 trial. Int J Chron Obstruct Pulmon Dis.

[10] Beeh K-M, Korn S, Beier J, Jadayel D, Henley M, D’Andrea P, Banerji D (2014). Effect of QVA149 on lung volumes and exercise tolerance in COPD patients: the BRIGHT study. Respir Med.

[11] O’Donnell DEE, Flüge T, Gerken F, Hamilton A, Webb K, Aguilaniu B, Make B, Magnussen H, Fluge T (2004). Effects of tiotropium on lung hyperinflation, dyspnoea and exercise tolerance in COPD. Eur Respir J.

[12] Casaburi R (2011). Activity promotion: a paradigm shift for chronic obstructive pulmonary disease therapeutics. Proc Am Thorac Soc.

[13] Troosters T, van der Molen T, Polkey M, Rabinovich RA, Vogiatzis I, Weisman I, Kulich K (2013). Improving physical activity in COPD: towards a new paradigm. Respir Res.

[14] Beeh KM, Watz H, Puente-Maestu L, de Teresa L, Jarreta D, Caracta C, Gil EG, Magnussen H (2014). Aclidinium improves exercise endurance, dyspnea, lung hyperinflation, and physical activity in patients with COPD: a randomized, placebo-controlled, crossover trial. BMC Pulm Med.

[15] Watz H, Krippner F, Kirsten A, Magnussen H, Vogelmeier C (2014). Indacaterol improves lung hyperinflation and physical activity in patients with moderate chronic obstructive pulmonary disease - a randomized, multicenter, double-blind, placebo-controlled study. BMC Pulm Med.

[16] Troosters T, Sciurba FC, Decramer M, Siafakas NM, Klioze SS, Sutradhar SC, Weisman IM, Yunis C (2014). Tiotropium in patients with moderate COPD naive to maintenance therapy: a randomised placebo-controlled trial. NPJ Prim Care Respir Med.

[17] Van Remoortel H, Raste Y, Louvaris Z, Giavedoni S, Burtin C, Langer D, Wilson F, Rabinovich R, Vogiatzis I, Hopkinson NS, Troosters T (2012). Validity of six activity monitors in chronic obstructive pulmonary disease: a comparison with indirect calorimetry. PLoS One.

[18] Hill K, Dolmage TE, Woon L, Goldstein R, Brooks D (2010). Measurement properties of the SenseWear armband in adults with chronic obstructive pulmonary disease. Thorax.

[19] Watz H, Waschki B, Boehme C, Claussen M, Meyer T, Magnussen H (2008). Extrapulmonary effects of chronic obstructive pulmonary disease on physical activity: a cross-sectional study. Am J Respir Crit Care Med.

[20] Watz H, Waschki B, Meyer T, Magnussen H (2009). Physical activity in patients with COPD. Eur Respir J.

[21] Miller MR, Hankinson J, Brusasco V, Burgos F, Casaburi R, Coates A, Crapo R, Enright P, van der Grinten CPM, Gustafsson P, Jensen R, Johnson DC, MacIntyre N, McKay R, Navajas D, Pedersen OF, Pellegrino R, Viegi G, Wanger J, Force AT (2005). Standardisation of spirometry. Eur Respir J.

[22] Quanjer PH, Stanojevic S, Cole TJ, Baur X, Hall GL, Culver BH, Enright PL, Hankinson JL, Ip MSM, Zheng J, Stocks J, (GLI) and the EGLFI, Initiative ERSGLF (2012). Multi-ethnic reference values for spirometry for the 3-95-yr age range: the global lung function 2012 equations. Eur Respir J.

[23] Roca J, Burgos F, Barberà JA, Sunyer J, Rodriguez-Roisin R, Castellsagué J, Sanchis J, Antóo JM, Casan P, Clausen JL (1998). Prediction equations for plethysmographic lung volumes. Respir Med Elsevier.

[24] Donohue JF (2005). Minimal clinically important differences in COPD lung function. COPD.

[25] O’Donnell DE, Lam M, Webb KA (1999). Spirometric correlates of improvement in exercise performance after anticholinergic therapy in chronic obstructive pulmonary disease. Am J Respir Crit Care Med.

[26] Oga T, Nishimura K, Tsukino M, Hajiro T, Ikeda A, Mishima M (2002). Relationship between different indices of exercise capacity and clinical measures in patients with chronic obstructive pulmonary disease. Heart Lung.

[27] Ries AL, Bauldoff GS, Carlin BW, Casaburi R, Emery CF, Mahler DA, Make B, Rochester CL, Zuwallack R, Herrerias C (2007). Pulmonary Rehabilitation: Joint ACCP/AACVPR Evidence-Based Clinical Practice Guidelines. Chest.

[28] Bolton CE, Bevan-Smith EF, Blakey JD, Crowe P, Elkin SL, Garrod R, Greening NJ, Heslop K, Hull JH, Man WD-C, Morgan MD, Proud D, Roberts CM, Sewell L, Singh SJ, Walker PP, Walmsley S (2013). British Thoracic Society Pulmonary Rehabilitation Guideline Development Group on behalf of the British Thoracic Society Standards of Care Committee. British Thoracic Society guideline on pulmonary rehabilitation in adults. Thorax.

[29] Spruit MA, Pitta F, McAuley E, ZuWallack RL, Nici L (2015). Pulmonary rehabilitation and physical activity in patients with chronic obstructive pulmonary disease. Am J Respir Crit Care Med.

[30] Spruit MA, Singh SJ, Garvey C, ZuWallack R, Nici L, Rochester C, Hill K, Holland AE, Lareau SC, Man WD-C, Pitta F, Sewell L, Raskin J, Bourbeau J, Crouch R, Franssen FME, Casaburi R, Vercoulen JH, Vogiatzis I, Gosselink R, Clini EM, Effing TW, Maltais F, van der Palen J, Troosters T, Janssen DJA, Collins E, Garcia-Aymerich J, Brooks D, Fahy BF (2013). An Official American Thoracic Society/European Respiratory Society Statement: Key concepts and advances in pulmonary rehabilitation. Am J Respir Crit Care Med.

[31] Casaburi R, Kukafka D, Cooper CB, Witek TJ, Kesten S (2005). Improvement in exercise tolerance with the combination of tiotropium and pulmonary rehabilitation in patients with COPD. Chest.

[32] Troosters T, Weisman I, Dobbels F, Giardino N, Valluri SR (2011). Assessing the impact of tiotropium on lung function and physical activity in GOLD Stage II COPD patients who are naïve to maintenance respiratory therapy: A study protocol. Open Respir Med J.

[33] Donaire-Gonzalez D, Gimeno-Santos E, Balcells E, de Batlle J, Ramon MA, Rodriguez E, Farrero E, Benet M, Guerra S, Sauleda J, Ferrer A, Ferrer J, Barberà JA, Rodriguez-Roisin R, Gea J, Agustí A, Antó JM, Garcia-Aymerich J (2015). Benefits of physical activity on COPD hospitalisation depend on intensity. Eur Respir J.

[34] Bateman ED, Ferguson GT, Barnes N, Gallagher N, Green Y, Henley M, Banerji D (2013). Dual bronchodilation with QVA149 versus single bronchodilator therapy: the SHINE study. Eur Respir J.

