# Supplementary material for: Effects of indacaterol/glycopyrronium (QVA149) on lung hyperinflation and physical activity in patients with moderate to severe COPD: a randomised, placebo-controlled, crossover study (The MOVE Study)
Source: BMC Pulm Med. 2016 Jun 14;16:95. doi: 10.1186/s12890-016-0256-7 (PMC4908762; doi:10.1186/s12890-016-0256-7)

Effects of indacaterol/glycopyrronium (QVA149) on lung hyperinflation and physical activity in patients with moderate to severe COPD: A randomized, placebo-controlled, crossover study (The MOVE Study)

Supplementary material

Henrik Watz,[1] Claudia Mailänder,[2] Monika Baier,[2] Anne Kirsten[1]

[1]Pulmonary Research Institute at Lung Clinic Grosshansdorf, Airway Research Center North, Member of the German Center for Lung Research, Woehrendamm 80, D-22927 Grosshansdorf, Germany

[2]Novartis Pharma GmbH, Nuremberg, Germany

Supplementary Figure 1. Raw mean peak inspiratory capacity after 21 days, overall and for each treatment period (full analysis set)

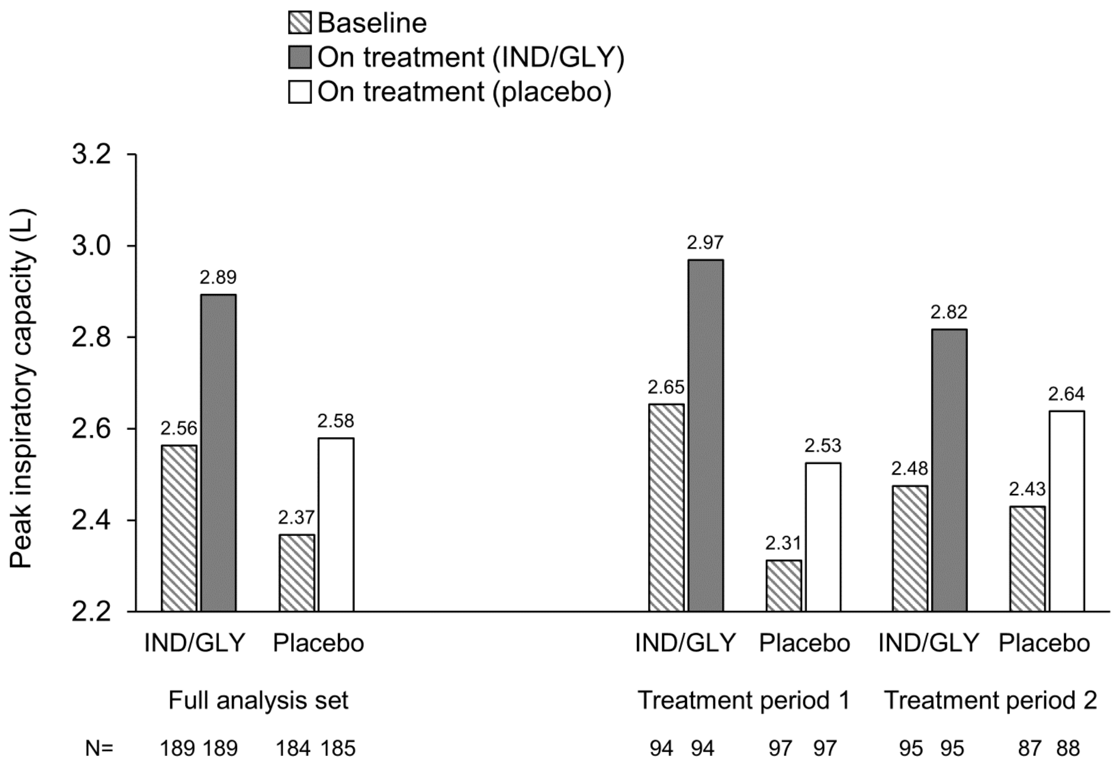

Supplementary Figure 2. Raw mean activity-related energy expenditure, overall and for each treatment period (full analysis set)

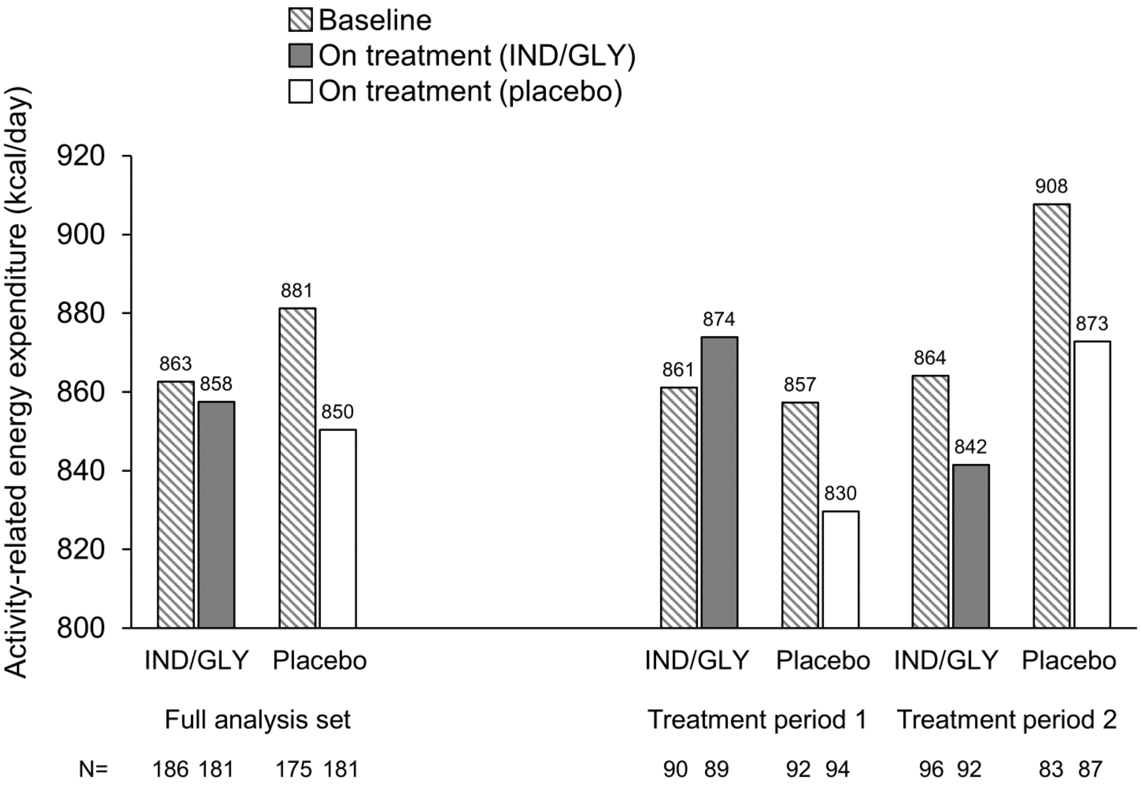

Supplementary Figure 3. LS mean change from baseline in activity-related energy expenditure, by median baseline FEV_1 and IC (full analysis set)

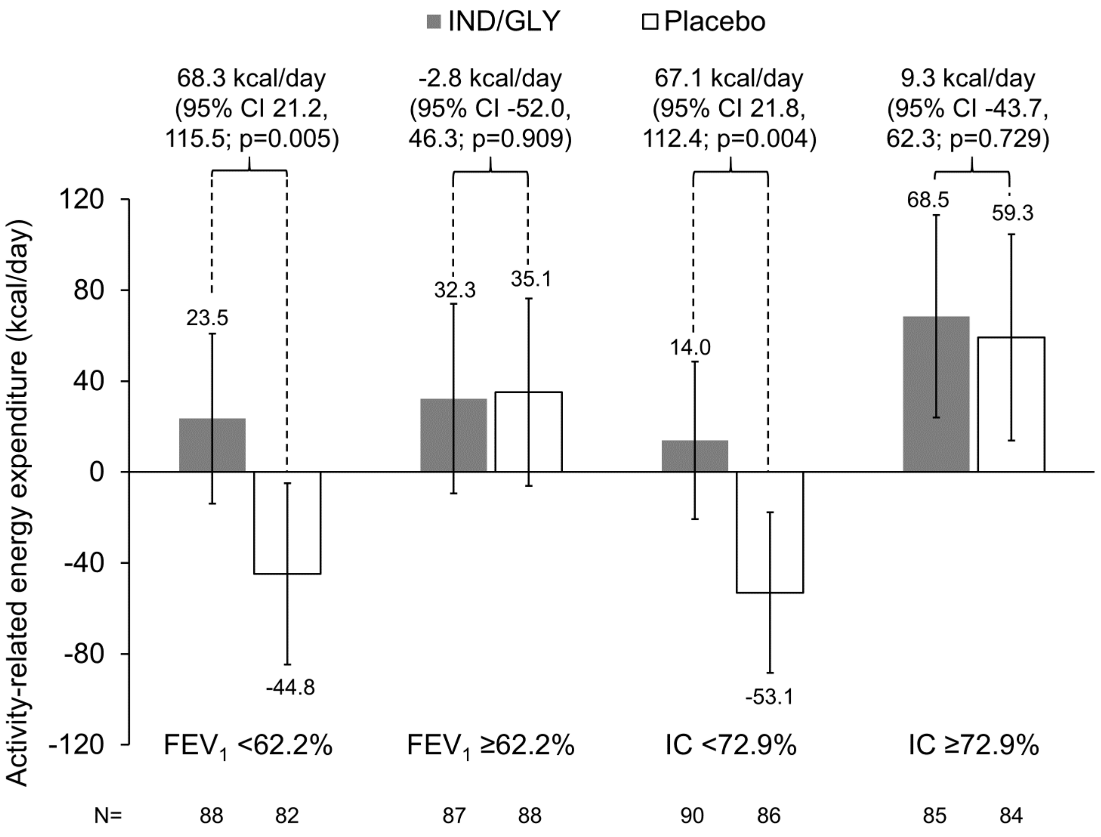

Supplement: Additional file 1: Figure S1. — Raw mean peak inspiratory capacity after 21 days, overall and for each treatment period (full analysis set). Figure presenting peak inspiratory capacity data, for the full analysis set and for treatment periods 1 and 2. Figure S2. Raw mean activity-related energy expenditure, overall and for each treatment period (full analysis set). Figure presenting activity-related energy expenditure, for the full analysis set and for treatment periods 1 and 2. Figure S3. LS mean change from baseline in activity-related energy expenditure, by median baseline FEV1 and IC (full analysis set). Figure presenting the change from baseline in activity related energy expenditure for four subgroups: Patients with baseline FEV1 < 62.2 % and ≥62.2 % (the median baseline value was 62.2 %); and patients with baseline IC <72.9 % and ≥72.9 % (the median baseline value was 72.9 %). (PDF 259 kb) [file 12890_2016_256_MOESM1_ESM.pdf]
